# Optimization of Three-Level Cervical Hybrid Surgery to Prevent Adjacent Segment Disease: A Finite Element Study

**DOI:** 10.3389/fbioe.2020.00154

**Published:** 2020-03-04

**Authors:** Chia-En Wong, Hsuan-Teh Hu, Meng-Pu Hsieh, Kuo-Yuan Huang

**Affiliations:** ^1^Department of Medicine, National Cheng Kung University Hospital, College of Medicine, National Cheng Kung University, Tainan, Taiwan; ^2^Department of Civil Engineering, National Cheng Kung University, Tainan, Taiwan; ^3^Department of Orthopedics, National Cheng Kung University Hospital, College of Medicine, National Cheng Kung University, Tainan, Taiwan

**Keywords:** cervical degenerative disc disease, ACDF, artificial disc replacement, hybrid surgery, finite element, biomechanics

## Abstract

Hybrid surgery (HS) allows surgeons to tailor fusion and arthroplasty in the treatment of multiple-level cervical disc degeneration. However, the decision making of selecting either ACDF or ADR for each level in three-level HS remains controversial and has not been fully investigated. This study was aimed to optimize three-level cervical hybrid constructs by systematically investigating their biomechanical properties and their effect on adjacent levels. A finite element model of cervical spine (C2–C7) was developed, and eight C3–C6 surgical models including six HS were constructed. The range of motion (ROM) in flexion, extension, lateral bending, and axial rotation under 2.0 Nm moments with 30 N follower load were simulated. The von Mises stress, strain energy at the adjacent intervertebral disc (IVD) and force at the adjacent facet were calculated. The ROM of the hybrid constructs and adjacent levels was close to that of the intact spine. HS with arthroplasty performed at C5-6 had better performance in terms of ROM reduction at the inferior adjacent level (C6-7). Moreover, C-D-D and 3ADR had best performance in reducing the von Mises stress and strain energy at C6-7. All HS reduced the facet burden at both C2-3 and C6-7 levels. However, the major drawback of HS revealed here is that the effect of C6-7 protection is at the cost of increased C2-3 IVD burden. In conclusion, we recommend C-D-D and 3ADR for patient with C3–C6 disc degeneration without predisposing C2-3 condition. C-C-D could be a good alternative with a lower medical cost. This analysis guides the decision making in three-level cervical HS before future cadaver studies or human clinical trials.

## Introduction

Anterior cervical discectomy and fusion (ACDF) surgery has been the gold standard treatment for patients with cervical disc degeneration who are unresponsive to conservative treatments (Persson et al., 1997; Hacker et al., 2000; Kaiser et al., 2002; Samartzis et al., 2005). However, surgical fixation and fusion markedly alters the biomechanics of the spinal segments, often resulting in reduced range of motion (ROM) in the operated level. This results in a compensatory hypermobility that can lead to accelerated degenerative effects in the motion segments adjacent to the fusion (Lee et al., 2014; Kong et al., 2016; Alhashash et al., 2018; Xu et al., 2018). Artificial disc replacement (ADR) is an alternative to ACDF to treat cervical degenerative disc disease while preserving motion at the operated level to prevent adjacent segment degeneration (ASD) (Pickett et al., 2005; Chang et al., 2007; Upadhyaya et al., 2012; Gao et al., 2015). However, the application of ADR has not been as widely used clinically as ACDF due to strict indications and higher medical cost (Ding and Shaffrey, 2012; Leven et al., 2017).

Hybrid surgery (HS) is a novel surgical strategy which allows surgeons to tailor ACDF and ADR at different levels to treat multiple level cervical disc degeneration and could potentially restore the physiological biomechanics of the cervical spine (Barbagallo et al., 2009; Shin et al., 2009; Hey et al., 2013; Lu et al., 2017). The design of cervical hybrid construct markedly affects its biomechanics as well as its impact on adjacent levels (Jia et al., 2014). However, due to the complexity of three-level cervical HS and lack of clinical and biomechanical evidence, the decision making of selecting either ACDF or ADR for each operated level in three-level HS remains controversial. Since there remains no guideline or standard for HS in the treatment of the three-level degenerative cervical disc disease, we designed a finite element study to investigate the biomechanical performance of different constructs of HS and determine which cervical hybrid construct provides better restoration of physiological motions and less burden to the adjacent segments.

Finite element analysis can be used to analyze the biomechanical performance of HS. Several previous finite element studies have investigated the characteristics of two-level HS. The finite element models evaluated the ROM in the levels adjacent to HS (Jia et al., 2014). Some studies compared the biomechanics with different hybrid construct in two-level cervical degenerative disc disease using different dynamic implants of various structure and material properties (Cho et al., 2010; Li et al., 2017; Mo et al., 2017). Cervical fusion was shown to change the ROM distribution of the cervical segments and lead to hypermobility as well as increased burden at the adjacent levels (Natarajan et al., 2000). HS is beneficial to motion preservation of the operated levels and produced a ROM of the entire operated and adjacent levels close to that of the healthy spine, resulting in less adverse effect on adjacent segments (Natarajan et al., 2000; Mo et al., 2017). Immobilization and segmental motion were achieved at the fusion level and arthroplasty level, respectively. These studies showed that HS is a safe and efficacious technique to benefit two-level cervical disc degeneration (Jia et al., 2014). Although emerging biomechanical studies have been conducted to evaluate different hybrid construct designs and different implants for two-level cervical disc disease, few studies discussed about three-level cervical diseases. Li et al. (2018) compared different HS using two stand-alone U-shaped dynamic implants in C3–C6 three-level cervical HS. The study showed that placing the dynamic implants in C3-4 and C4-5 levels leads to more compensation in terms of motion and facet stress and implies that the segmental motions should be taken into account when performing HS. However, the study only addressed constructs with one fused level and two dynamic implants. Different designs such as those with two ACDF level and one ADR has not been evaluated.

In the present study, we established finite element models of C2–C7 cervical segments and simulated six hybrid constructs for three-level cervical disc diseases at C3–C6. Flexion, extension, lateral bending, and axial rotation ROM of both the constructs and the adjacent levels were analyzed. Impact of each construct on adjacent levels including maximal von mises stress, strain energy, and force on facets were also compared. The objective of this study was aimed to compare and optimize the design of cervical hybrid constructs by systematically investigating their biomechanical properties and how they affect adjacent levels.

## Materials and Methods

### Generation of Cervical Finite Element Model

**FIGURE 1:**
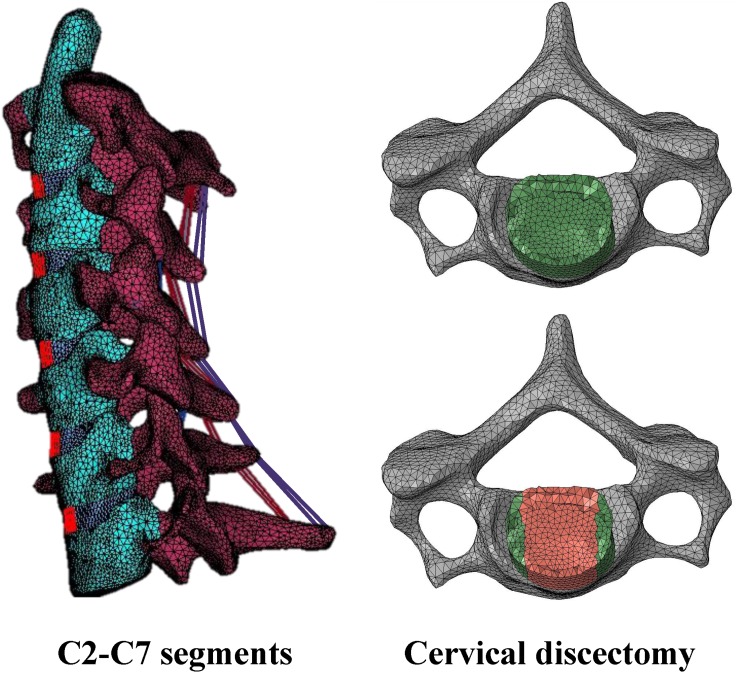
Finite element model of intact C2–C7 and cervical discectomy.

A three-dimensional finite element model of the C2–C7 cervical spine segments was developed from axial computed tomography images of the cervical spine obtained at 1-mm slice thickness (512 × 512 resolution, 16-bit, and a pixel size of 0.3516 mm × 0.3516 mm) from a resin spine model, which is cast from a cadaveric spine. The DICOM images were imported into the software 3D-DOCTOR software (Able Software Corp) to construct the geometric structure of C2–C7. The mesh structure was prepared using the preprocessing software Hypermesh 11.0 (Altair Technologies Inc) and then was imported into Abaqus 6.12 (Simulia Inc) to solve (Figure 1). This study adopted linear and isotropic material properties for cortical bone, cancellous bone, posterior bony elements, endplate, annulus fiber layers, annulus ground substance, and nucleus pulposus (Table 1).

**TABLE 1:** Material property and mesh type of the cervical finite element model.

**Component**	**Young’s modulus (MPa)**	**Poisson’s ratio**
**Annulus fiber**		
Inner laminate: Inner layer	360	0.30
Inner laminate: Middle layer	385	0.30
Inner laminate: Outer layer	420	0.30
Outer laminate: Inner layer	440	0.30
Outer laminate: Middle layer	495	0.30
Outer laminate: Outer layer	550	0.30
Annulus ground substance	4.2	0.45
Cancellous bone	100	0.20
Cortical bone	12000	0.30
Posterior bony elements	3500	0.25
Endplate	12000	0.30
Nucleus pulposus	1	0.49
ALL/PLL/LF/ISL/SSL	20/20/20/10/10	0.25
CoCrMo	200000	0.30
Artificial disc inlay (UMHWPE)	2400	0.30
Graft bone	100	0.20
Cage (PEEK)	3600	0.25

A vertebra consists of a cortical bone (thickness, 0.35 mm), cancellous bone, posterior bony elements, and endplates (thickness, 0.5 mm). A closed surface of cortical bones and endplates assigned to 3-node shell elements (S3R). Cancellous bone was assigned to 4-node solid tetrahedral elements (C3D4). The facet joints and the irregular posterior bony elements were modeled using solid tetrahedral linear elements (C3D4) according to the original geometry. The contact behavior of the facet joints was simulated with three-dimensional surface-to-surface contact with friction. To allow random motions including sliding, rotation, and separation, a finite sliding interaction was defined. The friction characteristic was modeled with a classic isotropic Coulomb friction model and a friction coefficient of 0.1 was assigned.

A disc is composed of a nucleus pulposus, annulus fiber layers and annulus ground substance (Supplementary Figure S1). The height of the IVD at C3-4, C4-5, C5-6 levels were 5.61, 5.75, 5.97, 5.85, and 6.04 mm, respectively. For the simulation of the IVD, annulus fibrosus was defined by an outer annulus fiber as the outer border, an inner annulus fiber as the inner interface between the annulus fibrosus and the nucleus pulposus, and the adjacent endplates as the superior and the inferior border. The annulus fibers were modeled with six layers of shell elements with the thickness of each layer of 1.5 mm. Annulus ground substance were defined between the two annulus fibers and the adjacent endplates and was modeled by solid tetrahedral linear elements (C3D4). Nucleus pulposus were defined by the inner annulus fiber and adjacent endplates and was modeled by solid tetrahedral linear elements (C3D4). Ligamentous complex including anterior longitudinal ligaments (ALL), posterior longitudinal ligaments (PLL), ligamentum flavum (LF), interspinous ligaments (ISL), and supraspinous ligaments (SSL) were modeled. The spinal ligaments were modeled as hyperelastic, tension-only, Truss elements (T3D2) to connect selected nodes on adjacent vertebrae. Material properties for the ligaments were derived from the ligament stiffness data from Goel et al. (1994; Table 2). The element types and number of elements used in the components of the spine are listed in Table 3.

**TABLE 2:** Properties of the ligaments in the present study.

**Ligament**	**ALL**	**PLL**	**LF**	**ISL**	**SSL**
Elastic modulus (small strain) (MPa)	7.8	10	15	10	8
Transition strain (%)	12	11	6.2	14	20
Elastic modulus (large strain) (MPa)	20	20	19.5	11.6	15
Cross sectional area (mm^2^)	63.7	20	40	40	30

**TABLE 3:** Element type and number of element in the intact cervical spine finite element model.

**Component**	**Element type**	**No. of elements**					
****	****	**C2**	**C3**	**C4**	**C5**	**C6**	**C7**
Cortical bone	S3R	2580	1991	2435	2732	2782	2485
Cancellous bone	C3D4	12645	11369	14792	16634	17048	13470
Endplate	S3R	710	828	883	865	960	867
Posterior bony elements	C3D4	16211	11427	12514	14259	11829	14782
		**C2-C3**	**C3-C4**	**C4-C5**	**C5-C6**	**C6-C7**	
			
Nucleus pulposus	C3D4	2017	2061	2130	2372	3273	
Annulus ground substance	C3D4	3120	2756	3442	3483	4330	
Annulus fiber	STRI3	1209	1044	1295	1419	1649	
Ligament		**ALL**	**PLL**	**LF**	**ISL**	**SSL**	
No. of elements	T3D2	25	20	20	15	10	

### Generation of Implant Models

**FIGURE 2:**
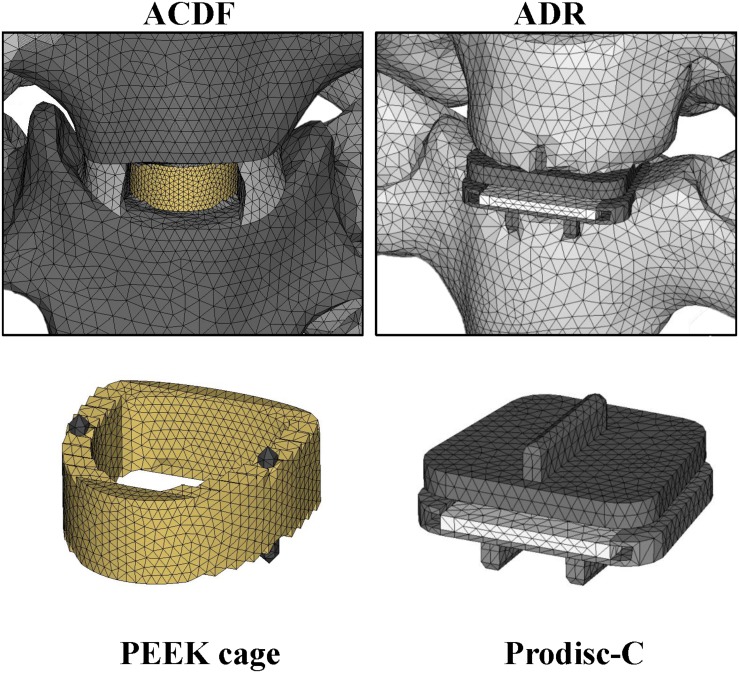
Finite element model of cervical surgical implants.

The implant model for artificial disc replacement was developed according to the Prodisc-C artificial disc (Synthes) implant (Figure 2). The primary dimensions (width, length, and heights) were 15, 12, and 6 mm, respectively. The superior and inferior implant plates composed of cobalt-chromium-molybdenum (CoCrMo) alloy with thickness of 2 mm, and the cores composed of Ultra-high molecular weight polyethylene (UHMWPE) with radius of 5 mm. The relative motion of the ball-and-socket core was modeled by surface-to-surface sliding contact with coefficient of friction of 0.5.

For fusion levels, implant model for cage was developed from the Solis Cervical PEEK Cage (stryker) (Figure 2). The primary dimensions (width, length, and heights) were 14, 12, and 6 mm, respectively. The cages composed of PEEK and the material properties of the implants were shown in Table 1. Three-dimensional structures of the artificial disc and cage implants were created in software Solidworks (Dassault Systemes SA) and mesh structures were prepared using software Hypermesh 11.0 (Altair Technologies Inc) and imported into Abaqus 6.12 (Simulia Inc) to solve.

### Surgery Simulation

Eight construct designs with six hybrid strategies were performed at C3–C6 level: (1) ACDF at all three level (C3–C6) (ACDF); (2) one ADR at C3/C4 level combined with two ACDF at C4–C5 and C5–C6 (Disc-Cage-Cage); (3) Cage-Disc-Cage; (4) Cage-Cage-Disc; (5) Cage-Disc-Disc; (6) Disc-Cage-Disc; (7) Disc-Disc-Cage; and (8) ADR at all three level (3ADR) (Supplementary Figure S2). For ACDF, discectomy was simulated with partial removal of the IVD included the nucleus pulposus, the anterior longitudinal ligament, the anterior annulus, the posterior longitudinal ligament, and the posterior annulus (Figure 1); for artificial disc replacement, the IVD was totally removed along with the anterior longitudinal ligament and the posterior longitudinal ligament. The end plate was shaped to fit the artificial disc implant in the ADR surgery. The interfaces of the implants and bone were assigned with tie constraint.

### Loading and Boundary Conditions

The loading condition consisted of a preload of 30N to simulate the head weight, and a moment of 2 M-m producing either flexion, extension, lateral bending, or axial rotation. The 30N preload was applied evenly using the follower load technique on the bilateral superior articular facets of C2 to simulate the weight of the head. To simulate cervical motions, the present study utilized a 2 N-m moment applied evenly on the C3–C6 segments, with a moment of 0.5 N-m on each segment. The rationale behind the design of the moment application is that the primary muscles producing cervical spine motions including longus coli (flexion), semiplinalis (extension), middle and posterior scalene muscles (lateral bending), and rotator (rotation) all had multiple insertions on multiple cervical segments. For each segment, a 0.5 N-m coupled moment was applied by application of evenly distributed force onto nodes over the anterior and posterior edges of the C3–C6 superior endplates (Supplementary Figure S3).

For all models, the boundary condition imposed were set with the nodes on the inferior endplate of C7 constrained in all directions. Finite sliding, surface-to-surface contact model with classical Coulomb friction was defined between the facets. The inferior and superior boundaries of the implants including cage and artificial disc were constrained to the endplates.

### Convergence Test

We used the intact model for convergence test and measured the displacement of a reference point on the top of the C2 dens under a 30 N preload on uniformly distributed on the C2 superior facets. Four different amounts: 222788, 208230, 175942, and 155899 elements were compared for their corresponding displacements. By setting the displacement of the C2 dens to 263379 elements as the reference value, the errors of the simulations with the total number of elements reduced were all within 6.6 percent. In this model, we selected a total of 222788 elements for intact model based on the small relative displacement error of 3.02%, with the element size ranging from 0.5 to 1.75 mm.

## Results

### Model Validation

**FIGURE 3:**
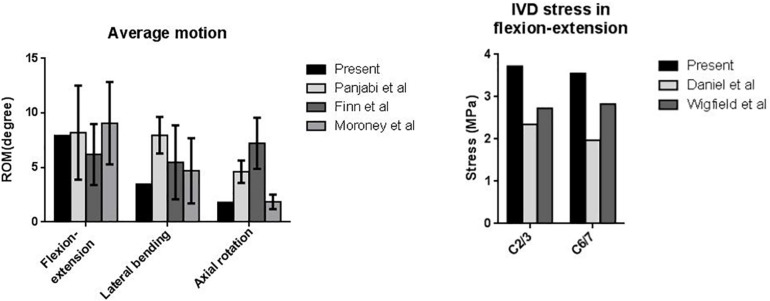
Comparison of C2–C7 cervical ROM and IVD stress with the literature.

For the validation of the finite element model, we compared the simulated ROM, IVD stress, and facet force of the intact model with those reported in the literature. First, the ROM of the intact cervical model was calculated and compared with the three *in vitro* experiments by Moroney et al. (1988), Panjabi et al. (2001), and Finn et al. (2009) (Figure 3). The average segmental ROM of the present intact cervical model values were as follows: flexion-extension, 7.93; lateral bending, 3.46; and axial rotation, 1.80. The results in flexion-extension and lateral bending was in good agreements with the literature. The ROM in rotation was smaller than the result of Panjabi et al. and Finn et al. but coincide with ones of Moroney et al. (1988), Panjabi et al. (2001), Finn et al. (2009). An extended explanation of the differences is made in the section “Discussion.”

Second, the model was compared with the *in vitro* profilometry results of the cervical IVD stress conducted by Wigfield et al. (2003) and Skrzypiec et al. (2007). In their experiments, the maximal IVD stress under sagittal flexion-extension was 1.9 Mpa to 2.9 MPa. The maximal IVD stress of the present model was 3.7 Mpa to 3.7 MPa (Figure 3).

Last, the model was compared with experiments by Chen et al. and Goel et al. for the facet contact force (Goel and Clausen, 1998; Chen et al., 2015). The ratio of the facet contact force to the applied axial loading was also calculated and the result was presented in Table 4. The ratio of the facet contact force to the preload was in good agreements with the literature.

**TABLE 4:** Facet contact force in the intact cervical segments.

**Intact segment**	**Present**	**Chen et al.**	**Goel et al.**
Preload (N)	30	73.6	73.6
Facet contact force (N)	4.64	11	8.4
Contact force/preload (%)	15.5	14.9	11.4

### Range of Cervical Motion and ROM Distribution

**FIGURE 4:**
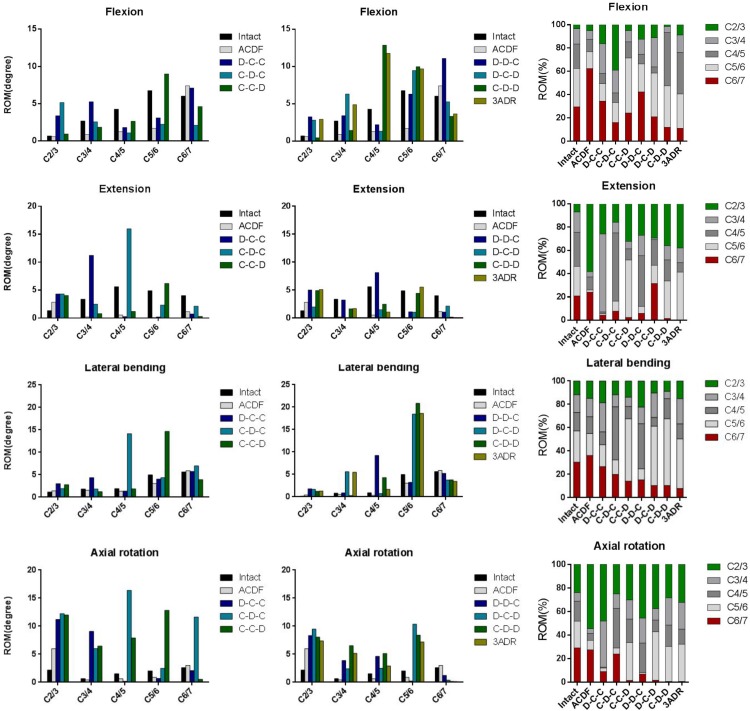
Range of motion and distribution in different cervical constructs.

The overall flexion, extension, lateral bending and axial rotation ROM of the intact and HS models were shown in Figure 4. Comparison between hybrid constructs with a single ADR and two cages showed compensatory hypermobility of C2-3 in extension, lateral bending and axial rotation for all three HS containing single ADR. In flexion, increase of ROM was more significant in D-C-C and C-D-C, while C-C-D had only mild increase in C2-3 flexion ROM. In motions of C6-7 segments, only the C-C-D construct had no compensatory hypermobility in all motions compared to the intact model. On the other hand, increased ROM was noted during flexion for D-C-C and during lateral bending as well as rotation for C-D-C.

For HS with two-level ADR and three-level disc replacements (3ADR), hypermobility of C2-3 lateral bending and rotation was noted in all constructs. Increased flexion ROM were also noted in D-D-C, D-C-D, and 3ADR, whereas increased extension ROM were noted in D-D-C, C-D-D, and 3ADR in C2-3 level. All constructs with two or three level ADR had decreased ROM in C6-7 level with the exception of D-D-C showed increased ROM of C6-7 in flexion.

The ROM distributions of the C2–C7 cervical segments were demonstrated in Figure 4.

C-C-D, D-C-D, C-D-D, and 3ADR showed similar ROM distributions to the intact spine in flexion, lateral bending and rotation while all constructs resulted in increased proportion in C2-3 extension.

**FIGURE 5:**
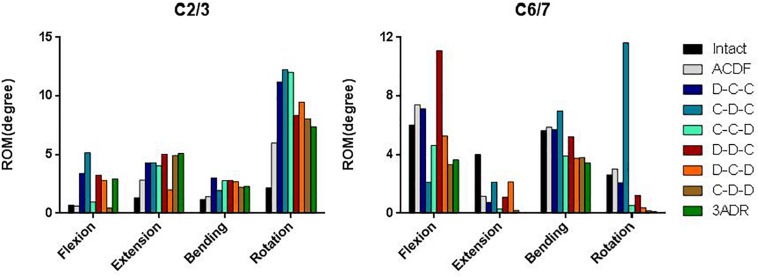
Comparison of adjacent level ROM in different cervical constructs.

The adjacent ROM of C2-3 and C6-7 were shown in Figure 5. Increased C2-3 ROM was observed in almost all motions in all constructs except flexion in C-D-D. For C6-7, better performance on prevention of adjacent hypermobility were observed in C-C-D, D-C-D, C-D-D, and 3ADR with reduced ROM in all motions compared to the intact spine model.

### von Mises Stress and Strain Energy on Adjacent Intervertebral Disc

**FIGURE 6:**
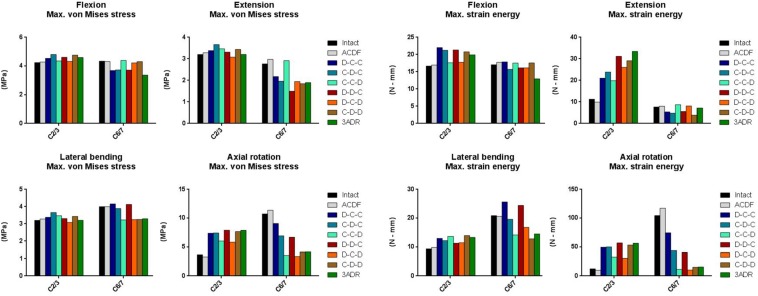
Maximal von Mises stress and strain energy at adjacent level in different cervical constructs.

The maximal von Mises stress and strain energy of each construct on adjacent IVD in cervical motions were shown (Figure 6). For all constructs, the maximal von Mises stress at the superior (C2-3) and inferior (C6-7) adjacent IVD under flexion, extension and lateral bending ranged approximately from 3.1 to 4.8 MPa and 1.9 to 4.4 MPa, respectively. The highest stress on both superior and inferior IVD occurred at axial rotation with highest value of 11.37 MPa in ACDF at C6-7 level, followed by 9.06 in D-C-C at C6-7 level. All HS decrease the stress at C6-7 level at the cost of increased stress in C2-3 intervertebral stress. The potential maximal stress at adjacent level for each construct and the corresponding motion were given in Supplementary Table S1.

The strain energy in C6-7 IVD was comparable between different constructs during flexion and extension. In C6-7 lateral bending, strain energy was increased in C-D-C and D-D-C; and were decreased in C-C-D, D-C-D, C-D-D, and 3ARD. In C6-7 level of intact and ACDF model, high strain energy accumulation during rotation was observed with 104.4 and 117.3 N-mm, respectively. The C6-7 rotational strain energy was reduced in all HS with better reduction in C-C-D, D-C-D, C-D-D, and 3ADR. For C6-7 IVD, the maximal strain energy of each HS was highest during rotation in D-C-C with 74.45 M-mm. The lowest maximal strain energy was observed during rotation in 3ADR, follow by rotation in C-D-D with 15.58 and 15.33 N-mm. On the other hand, the strain energies in the superior (C2-3) adjacent IVD were increased in all construct. The potential maximal strain energy at adjacent level for each construct was given in Supplementary Table S2.

### Facet Force at Adjacent Level

**FIGURE 7:**
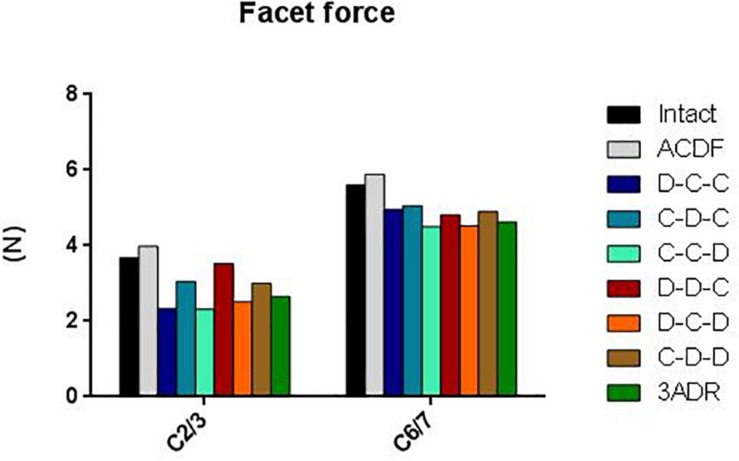
Total force at adjacent level facets during extension in different cervical constructs.

The load-sharing between the facet joints and the disc depends largely on spinal posture. At neutral position the cervical facets account for about 16 percent of axial weight transmission, this is increased up to 39 percent in extension posture (Adams and Hutton, 1980; Sharma et al., 1995). The total force on superior (C2-3) and inferior (C6-7) articular facets ranged from 2 to 4 N and 4.5 to 6 N, respectively (Figure 7). All HS reduced the total facet force at adjacent articular facets. Better reductions were observed in D-C-C, C-C-D, D-C-D, and 3ADR for C2-3; as well as C-C-D, D-C-D, and 3ADR for C6-7.

## Discussion

The present study simulated and compared the biomechanics of different C3–C6 cervical hybrid constructs with the aim to optimize HS in order to prevent ASD. An optimally designed surgical strategy would result in a construct which has a similar motion to that of an intact cervical spine while at the same time creating a similar IVD stress and facet contact force at the adjacent levels. A surgical construct that significantly alters the normal physiological response of the motion segment poses a risk of accelerated degeneration in the adjacent IVD as well as facets. In the present study, in contrast to applying the moment in the uppermost segment to simulate cervical spinal motion, we utilized an alternative strategy of moment application in which a 2 N-m moment was applied evenly on the C3–C6 segments, with a moment of 0.5 N-m on each segment. Based on the anatomical descriptions of Moore et al. (Chapter 4 and Chapter 8) (Moore et al., 2010), the muscles producing cervical spinal motions including longus coli (flexion), semiplinalis (extension), middle and posterior scalene muscles (lateral bending), and rotator (rotation) all had attachments on multiple levels of the cervical spine. As a result, a moment distributed evenly on the cervical segments may be more physiological. Similar concepts involving modeling the paraspinal muscles with multiple attachments were also applied by previous studies in the FE modeling of lumbar spine (Toumanidou and Noailly, 2015).

The FE model used in this study has been validated against experimental data of ROM, IVD stress, and facet force in the literature. Regarding the ROM, there are good agreements in the ROM in flexion-extension and lateral bending. We noted that the experimental data cited from the literature had high variability in axial rotation compared to flexion-extension and lateral bending. Several factors including the difference in the loading application and biologic variation between the specimens could also contribute to the difference between the results. Additionally, the assumption of isotropic materials in the FE model might also contribute to the difference of the biomechanics since the response of the spinal segments to moments in different plane may be different. However, the experimental results reported by Finn et al. (2009) had similar response in axial rotation ROM to the present study.

In regard to the IVD stress, the experimental data of stress profilometry cited from the literature were measured by pulling a miniature pressure transducer along the sagittal midline diameter of the disc (Wigfield et al., 2003; Skrzypiec et al., 2007). This technique allows the measurement of IVD stress along the trajectory of the pressure transducer but not the entire disc. It is quite probable that the area with maximal IVD stress is not on the trajectory of the pressure transducer and thus is not measured. This could explain the small difference of the IVD stress between the simulated result and the *in vitro* results from the literature.

The present study compared the ROM of different C3–C6 HS. Since cervical disc degeneration was found to be most prevalent in C5-6, and followed by C6-7 and C4-5, we aimed to prevent C6-7 ASD without causing significant burden in C2-3 (Constantoyannis et al., 2002). Our simulation showed that C-C-D, D-C-D, C-D-D, and 3ADR had best performance in reducing C6-7 ROM. This result suggested that performing ADR at the C5-6 level protects C6-7 against adjacent hypermobility.

Regarding the impact of HS on IVD in terms of stress and strain energy, since material failure occurs when von Mises stress surpasses tensile yield stress, the von Mises stress of the IVD implies the susceptibility of the IVD to acute failure (Doblaré et al., 2004). Acute failure of the disc might present clinically as ruptured annulus with protruding nucleus compressing the spinal cord or nerve root (Kelsey et al., 1984). On the other hand, cyclic strain energy during repetitive motion is related to fatigue of the material and therefore the strain energy of the IVD may be interpreted as the susceptibility to chronic disc degeneration (Pattin et al., 1996). Our simulation showed that although performing ADR at C5-6 effectively reduced the ROM at C6-7, it didn’t certainly reduce the von Mises stress and the strain energy. Our simulation suggested that C-D-D and 3ADR had better performance over C-C-D and hybrid surgeries also had an additional advantage in reducing facet burden. We recommend that C-D-D and 3ADR should be considered as the surgeries of choice when not contraindicated. However, one important drawback of HS should be noted, that is, effect of C6-7 protection was at the cost of increased ROM, von Mises stress and strain energy at C2-3. For patients with predisposing C2-3 disc degeneration that require C3–C6 surgery, the use of HS should be carefully evaluated and we recommend either performing ACDF or extend the construct to C2-3 level.

Taken together, our results suggested that of C-D-D, and 3ADR had best performance in the prevention of C6-7 ASD. In addition, construct with single ADR such as C-C-D could also be considered since it had similar benefits in C6-7 ROM reduction as those with two or three ADRs and C-C-D may be a good option for lowering the medical costs and for areas with limited medical resources. The major drawback of HS revealed by the present study is that increased C2-3 burden may preclude the use of HS in patient with predisposing C2-3 degeneration.

There are some limitations to the present study. First, ligaments were modeled as hyperelastic Truss elements. This simplification does not take into account the contact interaction between ligaments and vertebrae but has the advantage of avoiding unrealistic shearing forces in the ligaments and thus has a reduced computation time. Second, only linear isotropic material properties were used for the bony tissues and the IVD, which neglects the anisotropic properties of the materials. Third, the position and orientation of the implants are likely to have variations. Changes in position and orientation of the cage and the artificial disc may alter the ROM and stress distribution but this is very challenging to be taken into account since multiple factors in the real world such as surgical approach, anatomical variation, and surgeon’s preference may affect the positioning of the implant. Finally, perfect surface-to-surface contact with tie constraint were made between implants and bony endplate. This assumption would result in a smaller ROM than that of a clinical trial or *in vitro* study but had an advantage to simulate spinal adaptation to fusion and the implants compared to cadaveric experiments (Kothe et al., 2004). However, the main conclusions of the present study are based on a comparative analysis between the surgical models. The above-mentioned model simplifications are applied to all models and likely have minimal effect on the comparative differences between models. The biomechanical behavior of cervical hybrid construct must be evaluated first with finite element analysis and future clinical studies are warranted to evaluate if three-level HS could restore more physiological biomechanics of the spine without adverse consequences.

## Conclusion

In conclusion, the biomechanical behavior of three-level HS was compared in degenerative disc disease of C3–C6 using the finite element model of the C2–C7 cervical segments and we recommended C-D-D and 3ADR for patients with C3–C6 three-level disc degeneration since they preserved physiological ROM and reduced the burden on C6-7 adjacent segment. C-C-D is perhaps an alternative choice since it provides similar ROM to that of C-D-D and 3ADR but it results in slightly more stress in adjacent disc of C6-7. This analysis guides the decision making in three-level cervical HS, and future clinical trials are warranted to evaluate the feasibility of HS in three-level degenerative cervical disc disease.

## Data Availability Statement

The datasets generated for this study are available on request to the corresponding author.

## Author Contributions

C-EW and K-YH: conceptualization. C-EW, H-TH, M-PH, and K-YH: methodology. M-PH and H-TH: software. C-EW: validation. C-EW and K-YH: formal analysis. C-EW and K-YH: investigation. H-TH and K-YH: resources. C-EW and M-PH: data curation. C-EW and K-YH: writing – original draft preparation. C-EW, H-TH, M-PH, and K-YH: writing – reviewand editing. C-EW and M-PH: visualization. K-YH: supervision.

## Conflict of Interest

The authors declare that the research was conducted in the absence of any commercial or financial relationships that could be construed as a potential conflict of interest.
