# Supplementary material for: Optimization of Three-Level Cervical Hybrid Surgery to Prevent Adjacent Segment Disease: A Finite Element Study
Source: Front Bioeng Biotechnol. 2020 Mar 4;8:154. doi: 10.3389/fbioe.2020.00154 (PMC7064443; doi:10.3389/fbioe.2020.00154)
Supplement: Supplementary file 1 [file Data_Sheet_1.pdf]

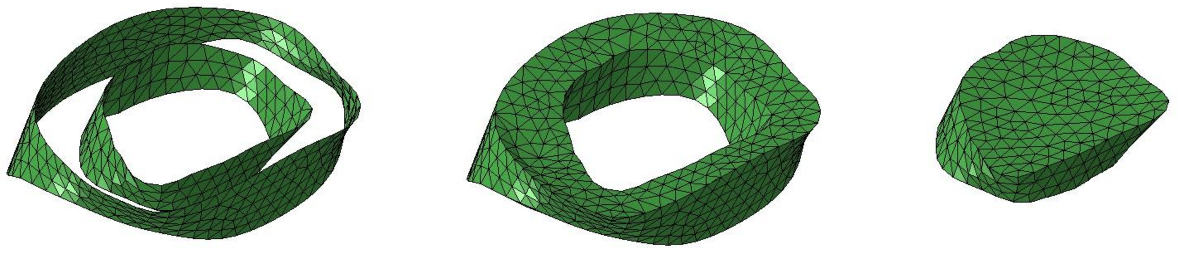

Annulus fiber layers **Annulus ground substance** **Nucleus pulposus**

Figure S1 Finite element model cervical IVD components

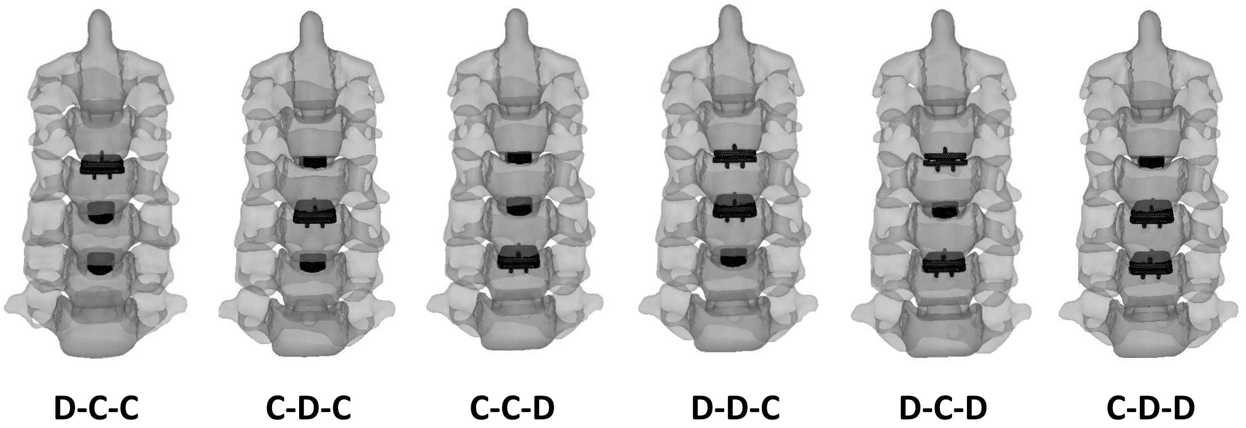

Figure S2 Schematic illustrations of cervical hybrid surgery

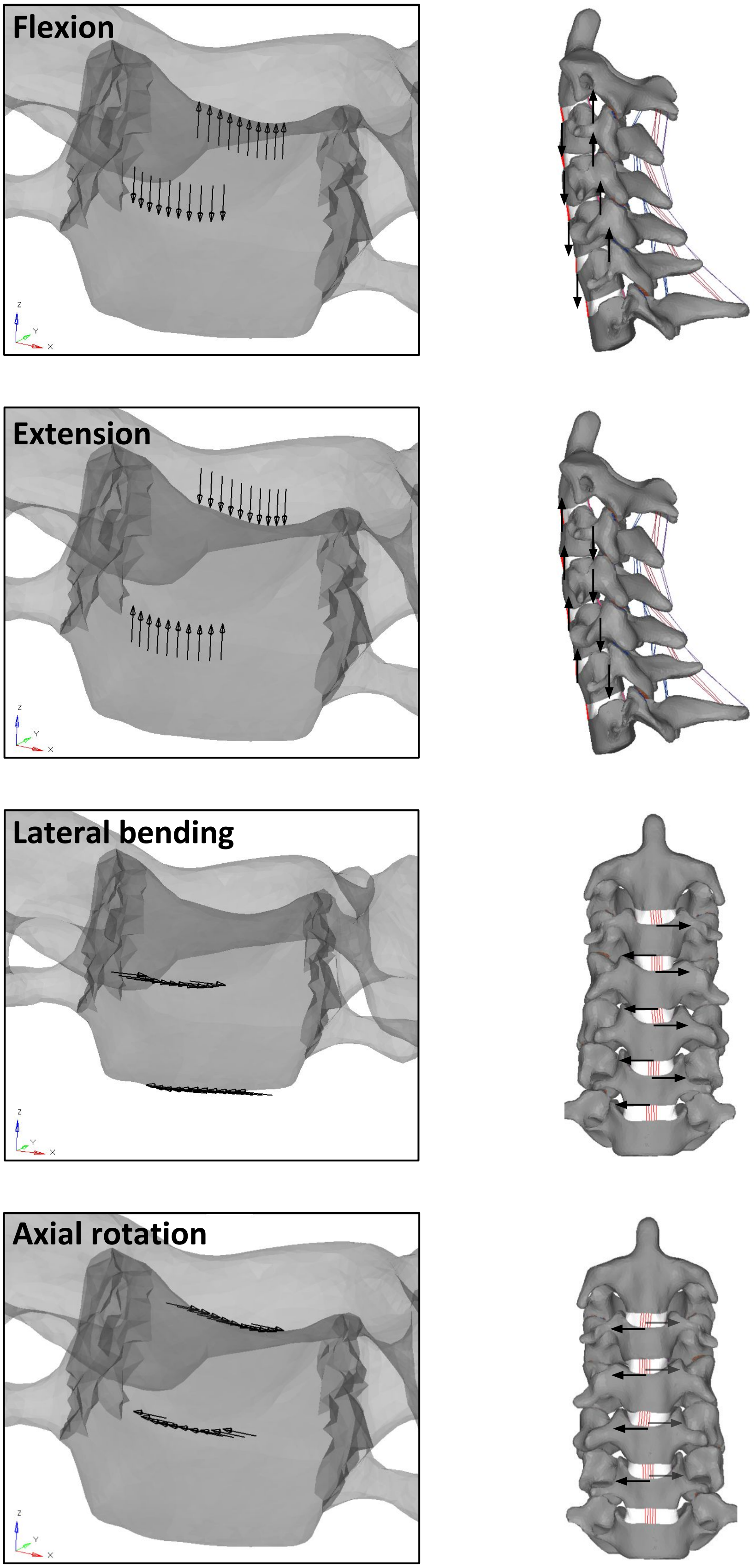

Figure S3 Moment application in cervical motions

Maximal stress at C2-3 and corresponding motion

construct	D-C-C	C-D-C	C-C-D	D-D-C	D-C-D	C-D-D	3ADR
stress (MPa)	7.38	7.43	6.02	7.91	5.82	7.65	7.88
motion	rotation	rotation	rotation	rotation	rotation	rotation	rotation

Maximal stress at C6-7 and corresponding motion

construct	D-C-C	C-D-C	C-C-D	D-D-C	D-C-D	C-D-D	3ADR
stress (MPa)	9.06	6.94	4.39	6.69	4.22	4.31	4.14
motion	rotation	rotation	flexion	rotation	flexion	flexion	rotation

Table S1 Maximal von Mises stress at adjacent level for each construct

Maximal strain energy at C2-3 and corresponding motion

construct	D-C-C	C-D-C	C-C-D	D-D-C	D-C-D	C-D-D	3ADR
energy (N-mm)	49.4	49.9	32.9	56.9	30.8	53.2	56.3
motion	rotation	rotation	rotation	rotation	rotation	rotation	rotation

Maximal stain energy at C6-7 and corresponding motion

construct	D-C-C	C-D-C	C-C-D	D-D-C	D-C-D	C-D-D	3ADR
energy (N-mm)	74.5	43.7	17.5	40.6	16.8	17.6	15.6
motion	rotation	rotation	flexion	rotation	bending	flexion	rotation

Table S2 Maximal strain energy at adjacent level for each construct
